# The effect of movement noise on internal estimations: possible implications for neural coding

**DOI:** 10.1186/1471-2202-12-S1-P381

**Published:** 2011-07-18

**Authors:** Miriam Zacksenhouse

**Affiliations:** 1 Faculty of Mechanical Engineering, Technion, Israel

## 

The development of Brain-Machine Interfaces (BMI) was motivated by the observed correlation between the neural activity of cortical motor neurons and the direction and speed of movement. However, these correlations were established during skilled arm movements, when the movement (process) noise, which affects the controlled movement, is relatively small (small co-variance matrix). In contrast, given the limited accuracy of BMIs, initial operation in brain control results in higher movement noise (larger co-variance matrix). Here we investigate the consequences of this change, and demonstrate that it may explain the changes in neural rate-modulations that accompany the transition from pole control to brain control in BMI experiments [1], and in particular the abrupt increase in their variability.

## Methods

Given the inherent delays in the sensory-motor system, it is generally assumed that the brain uses internal estimations of the state of the body, and in particular the hand, to control its movement. Optimal state estimation relies on internal prediction of the state, based on an internal model of the system, and on sensory measurements. The optimal integration of the internal prediction and sensory measurement depends on the relative size of their uncertainties: the measurement noise, which corrupts the measured sensory feedback, and the movement noise, which affects the actual movement and thus the deviation between the predicted and actual states. Thus, the internal estimation of the covariance matrices of these noise-sources affects optimal state estimation.

## Results

Using the framework of optimal state estimation, we show theoretically that under wide conditions (specified in the analysis), when the movement noise increases, the estimation process become more variable. The exact dependence between these two quantities is derived for a simple, 1 degree of freedom system, and compared with simulation results.

Neural activity is generated under the assumption that the neurons encode the estimated state, including the estimated position, velocity and speed. We demonstrate that the modulations of the simulated neurons increases as the movement noise increases. Finally, we simulate brain control by training a BMI filter and using its output to control the cursor. Assuming that the internal estimation of the movement noise is higher in brain control, the simulated neural activity demonstrates the same phenomena observed in the data: the percent neural modulations is higher in brain control without hand movements than in pole control, while the percent task-related modulations does not increase.
